# Polysaccharides derived from golden mushroom (*Cantharellus cibarius* Fr.) modulate gut microbiota and enhance intestinal barrier function to ameliorate dextran sulfate sodium-induced colitis in mice

**DOI:** 10.3389/fphar.2024.1498625

**Published:** 2024-12-18

**Authors:** Yamina Alioui, Hidayat Ullah, Sharafat Ali, Mujeeb Ur Rahman, Maroua Elkharti, Nabeel Ahmed Farooqui, Ata Ur Rehman, Muhammad Ilyas, Duaa M. Alsholi, Nimra Zafar Siddiqi, Muhsin Ali, Liang Wang, Yi Xin

**Affiliations:** ^1^ Department of Biotechnology, College of Basic Medical Science, Dalian Medical University, Dalian, China; ^2^ Department of Biochemistry and Molecular Biology, College of Basic Medical Science, Dalian Medical University, Dalian, China; ^3^ Multidisciplinary Neuroprotection Laboratories, Duke University School of Medicine, Durham, NC, United States; ^4^ Department of Medical Laboratories Sciences, Faculty of Allied Medical Sciences, Zarqa University, Zarqa, Jordan; ^5^ Stem Cell Clinical Research Center, National Joint Engineering Laboratory, Regenerative Medicine Center, The First Affiliated Hospital of Dalian Medical University, Dalian, China

**Keywords:** *Cantharellus cibarius* Fr., polysaccharides, gut microbiota, inflammatory bowel disease, dextran sulfate sodium, intestinal barrier

## Abstract

**Introduction:**

Inflammatory bowel disease (IBD), including ulcerative colitis, is marked by intestinal barrier disruptions, immune system dysregulation, and an imbalance in the gut microbiota. The golden chanterelle mushroom, *Cantharellus cibarius* Fr., a popular edible mushroom, has shown potential therapeutic benefits. This study examines the therapeutic potential of a crude polysaccharide extract obtained from *C. cibarius* Fr. (CCP) on intestinal barrier integrity, inflammatory cytokine levels, and gut microbiota composition in a murine model of colitis induced by dextran sulfate sodium (DSS).

**Methods:**

To induce colitis BALB/c mice were provided to consume autoclaved water with 3% DSS for 7 days, followed by 14 days of CCP supplementation. To assess the effects of CCP, histological analysis of colon tissue was performed, gene expression, inflammatory responses, tight junction proteins expression, gut barrier integrity, and cytokines levels were measured and analyzed and 16S rRNA sequencing were evaluated.

**Results and Discussion:**

CCP treatment alleviates colitis symptoms by improving body weight, and enhancing intestinal integrity through increased mucin-2 and tight junction protein expression. Additionally, CCP administration regulates the altered immune response by mitigating the expression of pro-inflammatory cytokines and upregulating anti-inflammatory cytokines. Furthermore, CCP supplementation effectively modulates DSS-induced dysbiosis as demonstrated by 16S rRNA sequencing results. These findings suggest that crude polysaccharides from the golden chanterelle mushroom, *C. cibarius* Fr., hold promise for treating colitis, via strengthening the intestinal barrier, regulating inflammatory responses, and reshaping the gut dysbiosis in a DSS-induced colitis model. CCP offers a novel approach for managing colitis, as a chronic inflammatory condition.

## Graphical Abstract

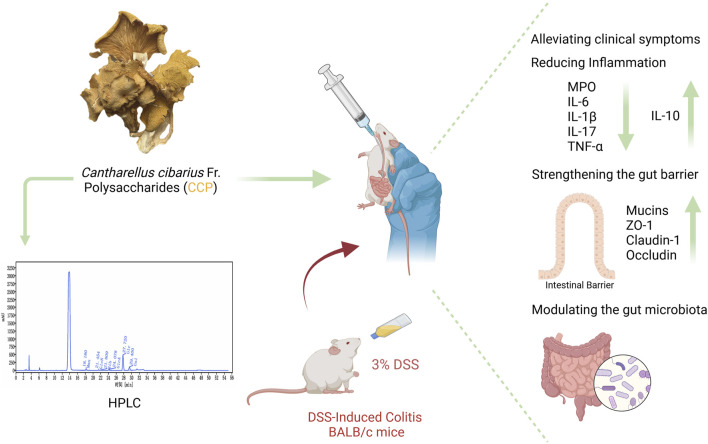

## 1 Introduction

Inflammatory bowel disease (IBD) encompasses two distinct clinical forms—ulcerative colitis (UC) and Crohn’s disease (CD). While CD affects any segment of the gastrointestinal tract, UC is predominantly affecting the colonic mucosa and manifests with different symptoms such as diarrhea and rectal bleeding (Xiang et al., 2021; Yang et al., 2021). No specific mechanism has been found to explain the precise etiology of IBD. Although a definitive mechanism for UC has yet to be elucidated, emerging evidence highlights the involvement of these different factors: genetic predisposition, environment, lifestyle, diet, immune function, and intestinal microbiome imbalance, collectively contributing to the occurrence of this significant health concern (Nunes et al., 2019; Alam et al., 2020). Emerging and metagenomic research has shed light on the gut microbiota’s role in the development and progression of ulcerative colitis revealing a potential association between dysregulation of immunological tolerance in the gut microbiota and the development of inflammatory bowel disease. Healthy individuals harbor a high richness of gut microbiota which is altered in UC patients. The integrity of the intestinal wall is compromised as a result of persistent mucosal inflammation since there is a limitation in the regenerative process of the epithelial barrier, it facilitates the translocation of intestinal contents, including bacteria and food antigens into the intestinal lumen thereby triggering the immune response based on the lamina propria and ultimately contribute to the pathogenesis of IBD (Alam et al., 2020; Duan et al., 2021; Nunes et al., 2018; Do et al., 2023). The prevalence of UC has been increasing in Western societies, such as North America, Europe, Australia, and New Zealand compared to Asia, Africa, and South America (Kotze et al., 2020) as a result it has been classified as a challenging public health concern by the World Health Organization (Duan et al., 2021; Do et al., 2023). Typically, the primary approach of UC involves the use of medications such as aminosalicylates, (e.g., 5-aminosalicylic acid and sulfasalazine). For more severe cases or when initial therapies fail, additional options may include oral corticosteroids, immunomodulators (e.g., thiopurines, methotrexate and, Janus Kinase inhibitors) and, biologic therapies, such as tumor necrosis factor (TNF) inhibitors and interleukin (IL)-12/23 inhibitors, targeting specific inflammatory pathways involved in IBD. These medications work by inhibiting pro-inflammatory cytokines, including TNF-alpha and IL-12/23, which play a crucial role in driving inflammation in the gut (Cai et al., 2021). These medications aim to manage UC, by reducing inflammation, maintaining remission and ultimately alleviating symptoms and enhancing patients’ quality of life. However, it is important to note that UC cannot be completely cured by existing treatment and, in some cases, it may fail to adequately control the disease. Consequently, severe cases may experience relapses or recurrences over time, which can lead to serious complications like toxic megacolon, perforation, uncontrollable bleeding, or the development of colon cancer, as patients with active UC are highly prone to developing colitis-associated colorectal cancer (Jin et al., 2017), in such instances, surgery may be necessary (Xu et al., 2004). In addition to the challenges of a high recurrence rate and associated complications, there are other serious problems associated with treating UC that cannot be ignored. Several side effects have been reported for the treatments of IBD, which can affect both the gastrointestinal system and the liver (Rogler, 2010; Kennedy et al., 2019). These inadequate treatment options available have spurred the question of alternative and novel therapeutic strategies that are devoid of side effects and one of the most suitable ones is the polysaccharides, which are attracting scientific interest as functional foods and therapeutic agents since it’s a natural compound, non-toxic with a wild range of pharmacological activities with and composed by multiple monosaccharide molecules, widely found in plants, animals, microorganisms and especially in mushrooms (Chen et al., 2019; Yu et al., 2018). Mushrooms have been consumed for centuries in various ways and due to their wide range of activities, contents and cost-effectiveness, fungal polysaccharides represent a promising solution for drug research (Fogarasi et al., 2021). *Cantharellus cibarius* Fr. mushroom found in coniferous forests, exhibits shades of orange or yellow hence the name Golden chanterelle in Britain, it is also known by various names in different countries, such as Girolle in France, Capo gallo in Italy, Yumurta mantari in Turkey, or simply Chanterelle as common name (Kozarski et al., 2015). It is highly prized for its delightful fruity aroma, and its exceptional flavor makes it widely recognized as one of the most popular and highly wild edible mushrooms in Europe and prevalent in North Africa, North America, and Asia, including the Himalayas and Yunnan in China (Drewnowska and Falandysz, 2015; Režić Mužinić et al., 2023; Keyhani and Keyhani, 2012). Despite its nutritional value, it is enriched with an abundance of nutrients including carbohydrates-based compounds, including polysaccharides, glucans, mannans, proteins, fatty acids, and low amounts of fats. Additionally, it has secondary metabolites like vitamins (D and A) phenolics, terpenoids, indole-based compounds, and other different microelements such as zinc, copper, iron, and selenium. It also showed some medicinal effects such as antioxidant, anti-inflammatory, antimicrobial, immunomodulatory, hypocholesterolemia, and antiviral properties (Drewnowska and Falandysz, 2015; Režić Mužinić et al., 2023; Keyhani and Keyhani, 2012; Sevindik, 2019; Kolundžić et al., 2017; Muszynska et al., 2016; Nowakowski et al., 2021; Dulger et al., 2004).

Based on existing information, *C. cibarius* Fr. is extensively collected and utilized as a wild mushroom in multiple countries. However, its effect on the treatment of colitis has not been disclosed. Therefore, the objective of this research was to examine the effects of the crude polysaccharide extract from *C. cibarius* Fr. (CCP) on colitis through modulation of the gut microbiota and inflammatory profile. This study hypothesized that *C. cibarius* Fr. derived polysaccharides could alleviate ulcerative colitis by modulating gut microbiota and restoring intestinal homeostasis.

## 2 Materials and methods

### 2.1 Chemical and reagents

Golden mushrooms (*C. cibarius* Fr.) were acquired from a (*Yunnan Congrong Economic and Trade Co., Ltd.* commercial supplier based in Yunnan, China), and dextran sulfate sodium (DSS) was provided by (Yeasen Biotechnology, Shanghai, China). All the antibodies were obtained from proteintech (Wuhan China) (Supplementary Table S1), Stool DNA extraction kit was procured from (MoBio Laboratories, Carlsbad, CA, United States). All additional reagents and chemicals utilized in this work were of analytical quality and acquired from reputable commercial supplier sources.

### 2.2 Preparation of crude polysaccharides from *Cantharellus cibarius* Fr. (CCP)

Upon receiving the *C. cibarius* Fr. mushrooms, fruiting bodies were separated, cleaned, and dried in a hot air oven at 60°C. Subsequently, the dried mushrooms were ground in a grinder to make a powder and passed through a 0.42 mm mesh sieve to obtain a pure fine powder. The powder was then subjected to two consecutive extraction procedures, each one using a different solvent-to-powder ratio. The first extraction was performed at a ratio of 1:30 (g/mL) for 4 h, subsequently, another extraction was carried out at 1:20 (g/mL) for 2 h, both extractions were conducted at 80°C. Following the extraction process, protein content was removed by treating the solution with 1.5% (v/v) TCA. The solutions were then adjusted to a pH of 7 using (NaOH) solution. The mixture was centrifuged at 5,000 rpm for 10 min, and the resulting supernatant was concentrated using a rotary evaporator. Following the concentration, four volumes of absolute ethanol were added to the solution and stored at 4°C overnight. Subsequently, the mixture was subjected to centrifugation at 5,000 rpm for 10 min, the supernatant was discarded and the remaining pellets were retained and considered as the crude polysaccharide extract from *C. cibarius* Fr., henceforth referred to as CCP. The CCP was then dried using a freeze-drying vacuum technique (Siddiqui et al., 2022). The protein content was evaluated and the CCP yield (%) was calculated as follows Equation 1:

$$\text{Yield }\%=\frac{W0}{W1}*100$$ (1)

### 2.3 Monosaccharide composition and carbohydrate content

The carbohydrate content was determined by the phenol-H_2_SO_4_ method, using D-glucose as standard (DuBois et al., 1956). The evaluation of the monosaccharide composition of crude polysaccharide (CCP) was done by high-performance liquid chromatography technique (HPLC).

### 2.4 Animal housing

Thirty-two male BALB/c mice, aged 4–5 weeks, and weighing 18 ± 2 g, were sourced from the SPF facility at Dalian Medical University and obtained ethical clearance from the institution’s ethics board under the approval number (202410368). The mice were distributed randomly into four different groups (n = 8 per group): normal control (NC), DSS-induced ulcerative colitis (DSS), low dose (150 mg/kg) *C. cibarius* Fr. Polysaccharide treatment (CCPL), and high dose (300 mg/kg) *C. cibarius* Fr. Polysaccharide treatment (CCPH) groups. Mice were acclimatized to standard laboratory conditions (temperature: 22°C ± 2°C, humidity: 50% ± 5%, light-dark cycle: 12 h) with access to standard chow and water.

### 2.5 Animal modelling and treatment protocol

**FIGURE 1:**
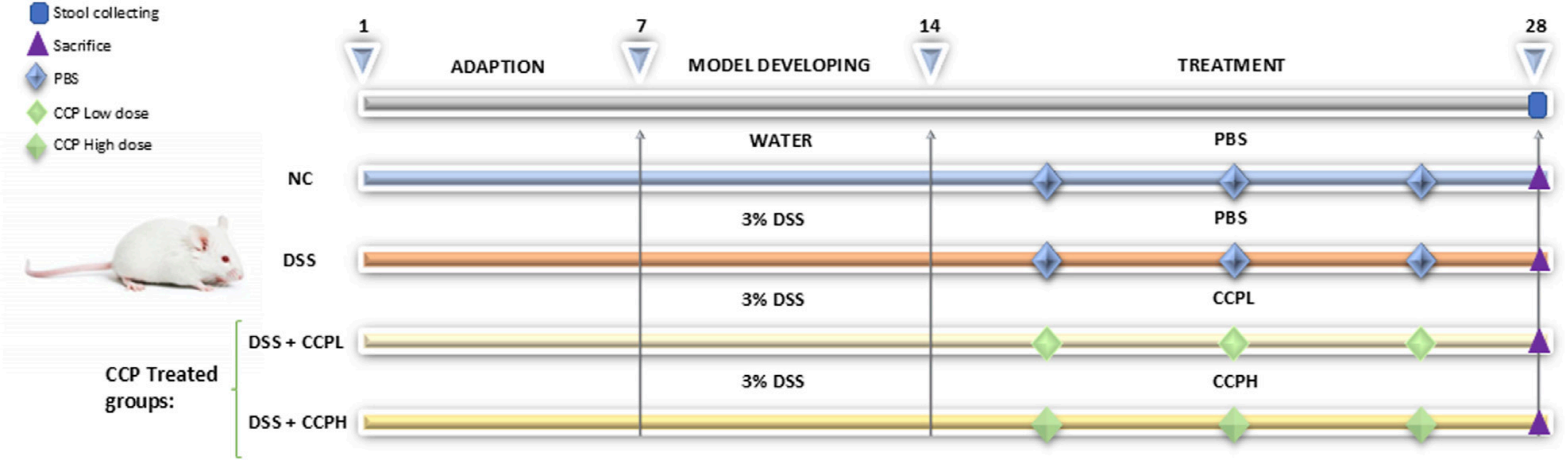
Schematic representation of the experimental design illustrating different steps at different time intervals in the study: the adaptation, the induction of colitis and treatment phases. The normal control group (NC, n = 8) was having access to normal water then administered PBS via gavage for the next 14 days. The colitis model (DSS, n = 8), low-dose treatment (CCPL, n = 8), and high-dose treatment (CCPH, n = 8) were administered 3% DSS in drinking water for 7 days to induce colitis. Following DSS treatment, the DSS group receive PBS by oral gavage for the next 14 days. The treatment groups CCPL and CCPH groups received low-dose and high-dose of CCP, respectively, by oral gavage for 14 days. Fecal samples were collected on day 28. Mice were euthanized on day 29.

To induce an ulcerative colitis (UC) model, mice were provided with 3% DSS dissolved in drinking water for 7 days. Following the establishment of the UC model, treatment supplementation was administered daily via gastric gavage for 14 days with the prescribed doses of crude polysaccharide freshly prepared in phosphate-buffered saline (PBS). The treatment groups included a low dose (150 mg/kg) for the CCPL group and a high dose (300 mg/kg) for the CCPH group, while the normal control group and UC model group received PBS only. A dose-ranging study was performed to identify the therapeutic window of CCP. Mice were administered various doses of CCP (50, 100, 150, 200, 300, and 400 mg/kg). The findings revealed that the lower doses 50 and 100 mg/kg showed no efficacy, while the 150 and 300 doses produced significant biological responses, warranting their selection for further research. The highest one, the 400 mg/kg dose did not demonstrate any significant improvement over the 300 mg/kg dose and was therefore considered unnecessary. Therefore, 150 and 300 mg/kg were selected for subsequent investigations. After a 29-day experimental period, fecal samples were collected individually and promptly stored at −80°C for microbiome analysis to evaluate the therapeutic efficacy and mechanisms of the administered treatments in the context of UC pathogenesis and progression. Following this, all mice were euthanized, tissue samples were collected and blood was centrifuged to collect serum, all were preserved at −80°C for biochemical assays, and molecular studies. Additionally, the distal colon was fixed in 4% buffered formalin for histopathological examination. The detailed study design is illustrated in Figure 1.

### 2.6 Body weight determination, disease activity index measurement, and organ indexing

Throughout the study period, the daily evaluation of colitis severity was assessed by the disease activity index (DAI) calculation. According to the Murthy scoring system (Yang et al., 2024; Murthy et al., 1993), based on a 4-point scoring system for three specific criteria: the percentage of the body weight loss (1 for 1%–5%, 2 for 5%–10%, and 3 for 10%–15%); stool consistency (0 indicated normal stools, and scores of 1 and 2 represented loose stools, while 3 and 4 indicated diarrhea); Regarding blood in the stool (with 1 for negative occult blood, 2 for positive occult blood, and 3 for gross bleeding). Finally, the DAI score was calculated as follows Equation 2:

$$\text{DAI}=1/3\;\left(\text{Weight loss score}+\text{Stool consistency score}+\text{Bleeding score}\right)$$ (2)

The length of colon tissue was measured from the cecum to the rectum and the weight of different organs: colon, small intestine, spleen, and thymus were taken individually for calculating organ index which consists of the ratio of organ weight (mg) to body weight (g).

### 2.7 Colonic histopathology

Following the sacrifice of the mice, and the fixation of the collected colon tissue samples in a 4% formalin solution at standard room temperature for 24 h. The fixed tissues were sliced, stained using hematoxylin and eosin, and dehydrated through a series of ethanol and xylene treatments. Finally, the stained tissue sections were mounted and sealed. After being prepared, the slides were examined under the microscope (Leica Microsystems, Wetzlar, Germany), and histological morphology, tissue regeneration, and inflammation were analyzed and evaluated using a scoring system (Table 1) (Nunes et al., 2019; Xu et al., 2020).

**TABLE 1:** Histological score: colon samples were assessed for two parameters, inflammation, and regeneration scores.

	Score	Significance
Regeneration	4	No tissue repair
	3	Surface epithelium not intact
	2	Regeneration with crypt depletion
	1	Almost complete regeneration
	0	Complete regeneration or normal tissue
Inflammation	3	Severe
	2	Moderate
	1	Slight
	0	None

### 2.8 Evaluating mucin production, goblet cell, and mucosal layer thickness

Goblet cells and the thickness of the mucus epithelium in colon tissue specimens were assessed via Periodic Acid-Schiff (PAS) staining. The process involved deparaffinizing tissue slides with xylene and subsequently rehydrating them through a sequence of ethanol solutions with decreasing concentrations. Following rehydration, periodic acid reagent was applied for 5 min at ambient room temperature, followed by three rinses in ultra-filtered water. Schiff reagent was then used for 10 min in a sealed container, and slides were washed under running water for 8 min. After counterstaining with hematoxylin and an additional 7-min wash under running tap water, slides were dehydrated in ethanol, clarified in xylene for transparency, and finally mounted using neutral balsam (cat-G8590, Solarbio). Alcian Blue staining (AB) at pH 2.5 was used to selectively detect epithelial acid mucins and non-sulfated and sulfated acid mucins. The slides were first deparaffinized and then hydrated as previously. Immersed in 1% aqueous alizarin blue acetate solution for 10 min for staining then washed three times for 6 min each. Followed by oxidation by immersion in 1% periodic acid solution for 5 min, washed with ultra-pure water two times, 6 min each. The slides were again immersed in Schiff’s solution for 10 min as staining and washed under tap water for 10 min. The slides were then dehydrated using a series of ascending alcohol solutions. Subsequently, slides were clarified, and the process was completed by mounting.

Immunohistochemistry (IHC) analysis was employed to examine the expression of Mucin-2 in the colon tissue samples. The prepared slides were subjected to a standard deparaffinization process using xylene, followed by rehydration through a decreasing gradient of ethanol concentrations. For antigen retrieval, the slides were immersed in a citrate buffer (10 mM, pH 6.0). The solution was heated using a microwave. After cooling, the slides were transferred to PBS (pH 7.4) and washed three times for 5 min each. To block endogenous peroxidase activity, the sections were incubated in an H_2_O_2_ solution (Sangon Biotech Co. Ltd. Shanghai, China) at room temperature for 25 min in the dark and then blocked in 5% BSA in PBS. The staining procedure was carried out following the manufacturer’s instructions (SP-KIT9720 immunohistochemistry staining kit, MXB Biotechnologies, Beijing, China). The slides were incubated with the primary antibody anti-mucin-2 (Proteintech, 27675-1-AP) at a dilution of 1:1,000 overnight at 4°C. Following this incubation, the slides underwent three washes with PBS, 10 min each, and then incubated with a secondary antibody at room temperature for 1 h. After the secondary antibody incubation, the slides were washed again, and 3,3′-diaminobenzidine (DAB) was applied for 5 min, followed by a thorough rinse with running tap water for 10 min. Hematoxylin counterstaining was performed for 5 min and the slides were once more washed for 10 min in running tap water to remove any excess stain. The slides were dehydrated through a graded series of ethanol and cleared with xylene. Finally, the slides were mounted with neutral balsam (cat-G8590, Solarbio). Each prepared slide was randomly observed in three different fields and examined under a microscope to assess the immunolabeled cells. ImageJ software was used for semi-quantitative analysis.

### 2.9 Immunofluorescent staining for tight junction protein

For immunofluorescence examinations of tight junction proteins (ZO-1, occludin, claudin-1) and nuclei staining in colon tissue sections, the slides were initially heated at 65°C for 2 h to enhance tissue adherence, followed by deparaffinization in xylene. Subsequent rehydration was achieved through a gradient of ethanol solutions (100%, 95%, 70%) and ultrapure water. Antigen retrieval was performed using citrate buffer (10 mM, pH 6.0) in a microwave oven, washed three times with PBS, then the tissue sections were blocked in 5% BSA in PBS and incubated overnight at 4°C with primary antibodies anti-ZO-1 (1:1,000, Proteintech, 21773-1-AP), anti-Occludin (1:400, Proteintech, 27260-1-AP) and anti-Claudin-1 (1:1,000, Proteintech, 13050-1-AP). Following primary antibody incubation, the slides were washed using PBS and subsequently incubated with FITC-conjugated Affinipure goat anti-rabbit (Proteintech) for 1 h at room temperature. Following another round of PBS washes, nuclei were stained with DAPI for 5 min. Finally, slides were mounted using a DAPI-containing medium and examined under a fluorescence microscope to assess the localization and expression patterns of the tight junction proteins and nuclei.

### 2.10 Myeloperoxidase activity (MPO) assay

To evaluate the myeloperoxidase (MPO) activity in colonic tissue, the supernatant from the colonic tissue homogenates was collected following homogenization of 100 mg of tissue in 900 µL of PBS in ice. The mixture was then centrifuged at 3,500 rpm for 15 min at 4°C^31^. Subsequently, the supernatant was collected for biochemical analysis. The detection of MPO activity in intestinal tissue was detected using an ELISA kit (Jiangsu Meibiao Biotechnology Co., Ltd.). According to the manufacturer’s guidelines, before the experiment, the ELISA kit’s reagents were pre-incubated at room temperature for 30 min. A 50 µL aliquot of the standard references were added to the standard wells and samples were added to the testing wells. Subsequently, 100 µL of HRP-conjugate was added to all wells, followed by a 60-min incubation at 37°C. The plate was washed five times with the washing buffer. Then, a colorimetric reaction was initiated by adding 50 µL of chromogen A and 50 µL of chromogen B to each well. After a 15-min incubation at 37°C protected from light, the reaction was stopped by adding 50 µL of stop solution. Finally, the absorbance values were measured at a wavelength of 450 nm within 15 min of the addition of stop solution, the standard curve was constructed and the concentration of each sample was calculated.

### 2.11 Quantifying colonic cytokine levels

As previously described, colon tissue was homogenized in PBS on ice to collect the supernatant, to assess the anti-inflammatory effect of CCP in the context of DSS-induced colitis. After homogenization, the mixture was centrifuged, and the supernatant was collected for the detection of cytokines. ELISA kits (Jiangsu Meibiao Biotechnology Co., Ltd.) were used according to the previously described protocol of the manufacturer to measure the following pro-inflammatory cytokines IL-1β, IL-17, TNF-α, and IL-6, as well as the anti-inflammatory cytokine IL-10 in the colon sample supernatants.

### 2.12 Determination of intestinal mRNA

To evaluate the therapeutic impact of CCP on DSS-induced intestinal inflammation, mRNA levels of pro-inflammatory (IL-6 and TNF-α) and anti-inflammatory cytokine (IL-10) were measured. Total RNA from colon tissue was extracted using TRIzol reagents (Thermo Fisher Scientific Waltham, MA, United States), quantified with a NanoDrop ND-1000 spectrophotometry, and stored at −80°C until further use. cDNA was synthesized using 1 ug of RNA, according to the manufacturer’s instructions using HiScriptR II Q RT SuperMix for qPCR (Vazyme biotech Co., Ltd.). Gene expression analysis was carried out with ChamQ SYBR qPCR MasterMix and Bioer Light Gene 9,600 analyzers (Hitech, Binjiang, District, Hangzhou, 310,053, China). The PCR protocol included an initial denaturation at 95°C for 10 min, followed by 40 cycles at 95°C for 25 s and 60°C for 1 min. Relative gene expression was analyzed using the 2^−ΔΔCt^ method, the Gene 9,660 system software, and GraphPad Prism for statistical analysis, with β-actin as the reference gene. Supplementary Table S2 shows the primer sequences.

### 2.13 Stool DNA extraction and 16S rRNA gene sequencing

For the stool DNA extraction, the PowerMax DNA Isolation Kit (MoBio Laboratories, Carlsbad, CA, United States) was used to extract total fecal microbial genomic DNA from all samples, as per manufacturer’s instructions. The procedure for bacterial DNA extraction from mouse feces involves several key steps. The procedure begins with sample preparation, where 200–500 mg of fecal samples are weighed and placed into a tube to ensure accurate dosing for extraction. The subsequent cell lysis step employs a combination of enzymatic and chemical treatments. Lysozyme is used to degrade bacterial cell walls, while a chaotropic salt solution disrupts cell membranes, facilitating the release of genomic DNA. Protease and buffer treatment is then applied to further digest proteins, enhancing the overall yield and purity of the DNA. Following lysis, a buffer solution is utilized to remove proteins and other contaminants, ensuring a cleaner DNA extraction. The lysate is then subjected to a series of centrifugation and purification steps, utilizing spin-column technology. Binding buffers are applied to facilitate DNA binding to the column during this purification process. Contaminants are effectively removed through multiple wash steps using specialized buffers. Finally, the purified and concentrated DNA is eluted from the column membrane with an elution buffer. The extracted DNA is stored at −80°C for subsequent molecular analyses. After extraction, DNA quantification was performed using a NanoDrop ND-1000 spectrophotometry, and DNA quality was evaluated by electrophoresis on a 1% agarose gel. To analyze the gut bacterial community, PCR amplification was performed targeted the 16S rRNA V3-V4 region using primers 341f (CCT​ACG​GGA​GGC​AGC​AG) and 518r (ATT​ACG​CGG​CTG​CTG​G). The PCR protocol began with an initial denaturation at 98°C for 30 s, followed by 25 cycles consisting of denaturation at 98°C, annealing at 58°C, and extension at 72°C, each step lasting 15 s. A final extension was performed at 72°C for 1 min. Sequencing of the amplicons was carried out using the Illumina NovaSeq 6,000 platform, with the first sample set sequenced at Sangon Biotech [(Shanghai) Co., Ltd., China.], and subsequent bioinformatics analysis employed the Quantitative Insights into Microbial Ecology (QIIME) software package. Alpha diversity indices including Chao1, Shannon, Ace, and Simpson were calculated to evaluate species richness and evenness. Operational taxonomic units (OTUs) were identified, and a Venn diagram was constructed to visualize unique and shared OTUs among sample groups. Additionally, beta diversity was analyzed through weighted UniFrac distance metrics: principal coordinates analysis (PCoA), principal component analysis (PCA), and non-metric multidimensional scaling (NMDS) to assess community structure differences. Finaly, LEfSe (Linear Discriminant Analysis Effect Size) and Linear Discriminant Analysis (LDA) was employed to identify microbial taxa contributing to differences between experimental groups and to pinpoint key biomarkers. This approach provided comprehensive insights into the composition and dynamics of the gut microbiota.

### 2.14 Statistical analysis

For this study, statistical analysis was conducted using GraphPad Prism 9 software. For comparison among more than two groups with equal standard deviation, ordinary analysis of variance (ANOVA) followed by Tukey’s multiple comparison tests was employed. A *p-*value less than 0.05 was considered statistically significant, and all the data was presented as mean ± standard deviation (SD). Additionally, for 16s rRNA sequencing data. LEfSe analysis utilized Kruskal–Wallis and Wilcoxon tests, while phenotypes and OUT statistical analysis employed the Mann-Whitney test.

## 3 Results

### 3.1 Chemical analysis and characterization of *Cantharellus cibarius* Fr. polysaccharide (CCP)

**FIGURE 2:**
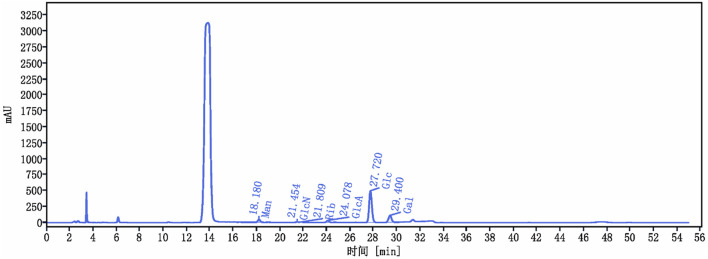
*Cantharellus cibarius* Fr. Polysaccharide (CCP) Characterization using HPLC. HPLC chromatogram of CCP. The retention times of the individual monosaccharides are indicated. Peaks correspond to 1: Mannose (Man), 2: Glucosamine (GlcN), 3: Ribose (Rib), 4: Glucuronic acid (GlcA), 5: Glucose (Glc), and 6: Galactose (Gal).

The yield of *C. cibarius* Fr. polysaccharide was 14% with 2.1% protein content and 45 mg/mL of carbohydrates using D-Glucose as standard. The crude polysaccharide CCP was analyzed using HPLC (High-Performance Liquid Chromatography), and the results indicated that it is composed of the following monosaccharides: Mannose, Ribose, Glucuronic Acid, Glucose, and Galactose, as presented in Table 2. The HPLC spectrum is presented in Figure 2.

**TABLE 2:** The monosaccharide composition of *Cantharellus cibarius* Fr., crude polysaccharides.

Components	Concentration (mg/kg)	Percentage (%)
Mannose	16,769.52	3.372662479
Ribose	5,820.95	1.170701347
Glucuronic acid	17,996.19	3.619368639
Glucose	404,860.95	81.42506972
Galactose	51,771.43	10.41219781

### 3.2 *Cantharellus cibarius* Fr. polysaccharide (CCP) alleviates inflammatory symptoms

**FIGURE 3:**
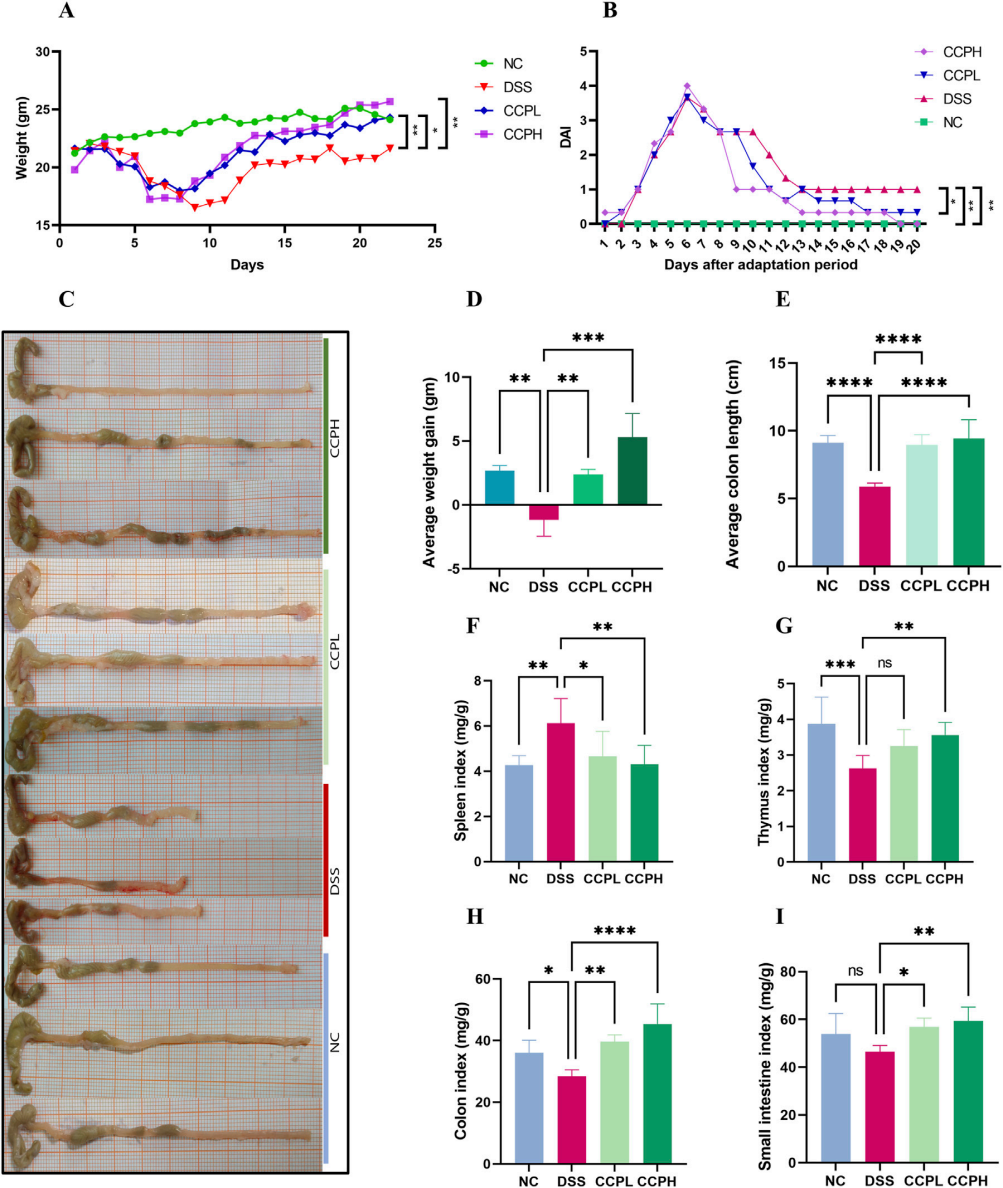
CCP alleviates colitis symptoms. **(A)** average weight change curves; **(B)** Disease Activity Index scores; **(C)** colon length; **(D)** Average weight gain; **(E)** Average colon length; **(F)** Spleen Index; **(G)** Thymus Index; **(H)** Colon Index; **(I)** Small intestine Index. Results: ns (not significant), *p < 0.05, **p < 0.01, ***p < 0.001; ****p < 0.0001.

The Ulcerative Colitis (UC) model was induced in mice by administering 3% of DSS in their drinking water. Following a 7-day modeling period, mice underwent a 14-day treatment phase with CCP. Throughout both phases, various clinical parameters associated with UC were monitored daily, including body weight, weight gain, rectal bleeding, and fecal consistency, as illustrated in Figures 3A, B, D. Figure 3A shows that during DSS treatment, all DSS-induced groups exhibited decreased body weight, accompanied by severe diarrhea and rectal bleeding. These symptoms collectively validated the successful establishment of the DSS-induced UC model. Interestingly, during the recovery phase, all mice showed weight gain (Figure 3A). However, the DSS group exhibited significantly less weight gain in comparison with the normal control group (NC) (*p* < 0.01). In contrast, both CCPL and CCPH treatment groups showed weight gain similar to NC and significantly higher than the DSS group (*p* < 0.01 and *p* < 0.001, respectively) (Figure 3D). The severity of ulcerative colitis was evaluated using the Disease Activity Index (DAI) scores (Figure 3B). The data revealed increased DAI scores in the DSS, CCPL, and CCPH groups, indicating symptoms such as weight loss, bloody stools, and diarrhea. In contrast, the NC group maintained a stable DAI score of 0 throughout the experiment. Importantly, upon introduction of CCP treatment following DSS removal, noticeable improvements were observed in the treatment group compared to the DSS group. Specifically, DAI scores decreased to 0 in the CCPH group by the end of the experiment, indicating a noticeable amelioration of disease symptoms. On the final day, there were significant differences in DAI scores between the DSS group compared to NC (*p* < 0.01), as well as CCPL and CCPH groups compared to DSS group (*p* < 0.05 and *p* < 0.01, respectively). These findings underscore the effectiveness of CCP treatment, particularly at higher doses, in mitigating the severity of ulcerative colitis induced by DSS, as evidenced by the substantial reduction in DAI scores.

### 3.3 CCP effects on organ indices and colon length

In order to assess the severity of the colitis model, the colon length, histological assessment, and organ indexing are evaluated. As shown in Figures 3C, E, a notable shrinkage in the colon of the model group compared to the normal control group is observed (*p* < 0.0001). Nevertheless, CCP administration led to a significant increase in colon length in both treatment groups (*p* < 0.0001). Furthermore, colon, small intestine, thymus, and spleen indexing, which represents the ratio of the organ weight to the body weight, served as an indicator of the inflammation severity. The data indicated a significant increase in the spleen index, while the indices for the thymus, small intestine, and colon were notably lower in the model group in comparison to the normal group. Thymus atrophy and spleen enlargement are hallmarks of dysregulated immune responses associated with inflammatory conditions. These pathological alterations were substantially mitigated with CCP (Figures 3F–I) in CCPL and CCPH groups. CCP treatment in a dose-dependent manner, significantly attenuated spleen enlargement and thymic atrophy compared to the DSS group. These observations indicate that treatment with CCP may have potent immunomodulatory properties that could be significantly beneficial in the context of colitis.

### 3.4 CCP improves histological changes and attenuated DSS-induced colon injury in mice

**FIGURE 4:**
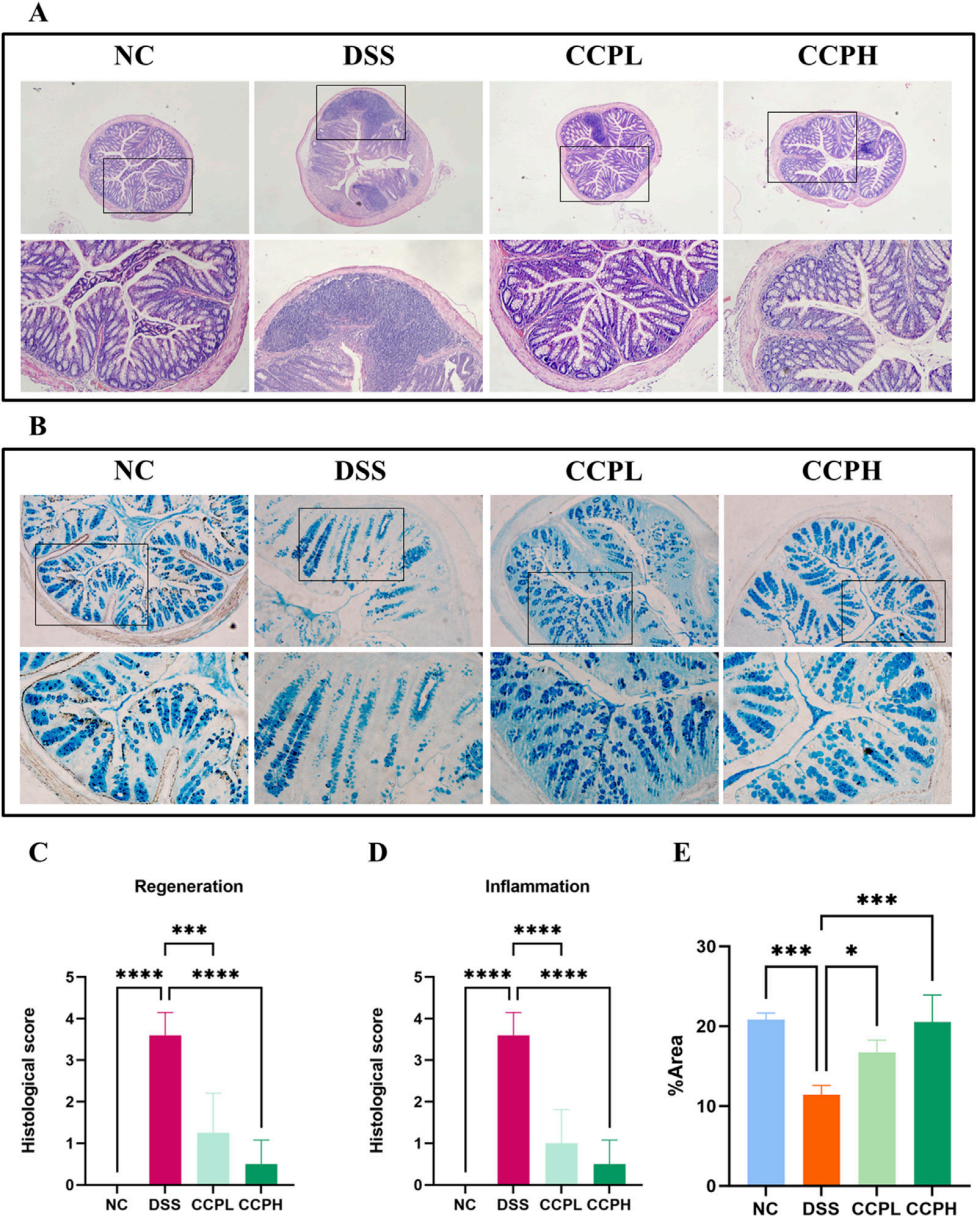
CCP alleviates DSS-histological damages in colon tissue. **(A)** Hematoxylin and Eosin (H&E) staining magnification (upper ×10) (lower ×20); **(B)** Alcian Blue staining (AB) staining indicates acid mucins secreted by goblet cells. Magnification: (upper ×10) (lower ×20); **(C)** Histological score (Regeneration); **(D)** Histological score (Inflammation); **(E)** Quantification Graph of AB staining. Results: *p < 0.05, ***p < 0.001; ****p < 0.0001.

Hematoxylin and Eosin (H&E) staining was employed and then analyzed by assessing histological scores to evaluate further the effect of CCP on the severity of inflammation and the histopathological changes in colon morphology. The staining observation revealed that the DSS group exhibited characteristic pathological alterations of UC: epithelial damage, depletion of mucus-producing goblet cells, crypt abscess formation, and infiltration of immune cells, compared to the NC group, where the colon appeared normal, with healthy architecture and an intact epithelium (Figures 4A, C, D). Nonetheless, in the two treatment groups, the staining shows the partial recovery of colon damage of CCP by restoring the overall characteristics of the colonic tissue, including crypt loss, enhancement of goblet cell production, and decreased inflammatory cell infiltration in a dose-dependent manner. Histopathologic scores assessed the inflammation and recovery of the colon and revealed a significant elevation in the score of the model group compared to the normal group, with a considerable reduction of this score in CCPL and CCPH compared to the model group. These results confirmed the potency of CCP in mitigating the histopathological alterations of DSS-induced ulcerative.

### 3.5 CCP induces epithelium repair by enhancing the expression of mucins

**FIGURE 5:**
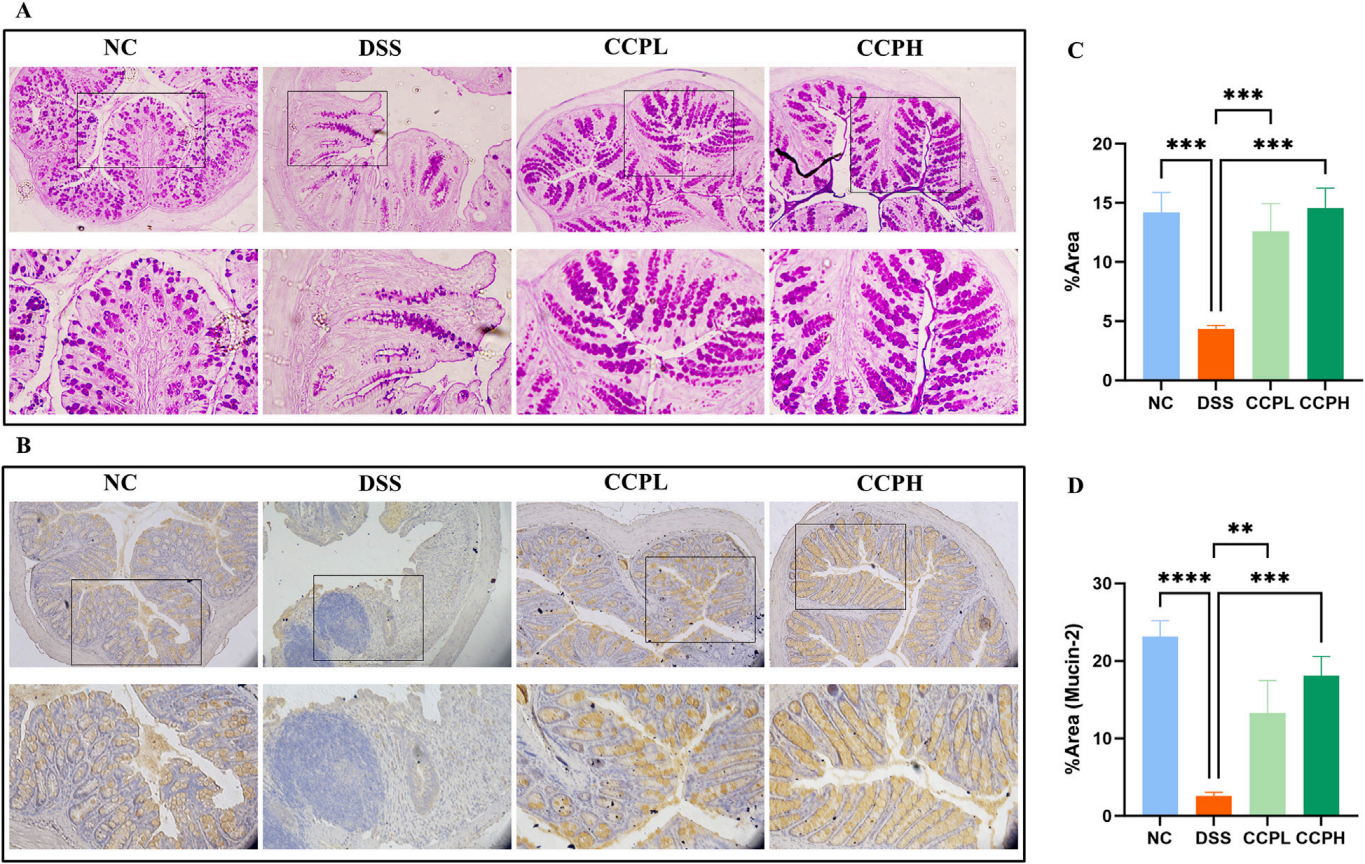
CCP improves Mucin expression in colon tissue. **(A)** Periodic Acid-Schiff (PAS) staining indicates the neutral mucins in the goblet cells; **(B)** Immunohistochemistry (IHC) staining detects the expression of Mucin-2 protein in colon tissues, Magnification: (upper ×10) (lower ×20); **(C)** Quantification Graph of PAS staining; **(D)** Quantification Graph of IHC staining (Mucin-2). Results: **p < 0.01, ***p < 0.001; ****p < 0.0001.

The intestinal mucus layer protects the epithelial barrier of the colon; it is formed by acidic and neutral mucins, which are secreted by a large amount of goblet cells. Alcian blue (Figures 4B–E) and Periodic Acid-Schiff (PAS) (Figures 5A, C) were used to investigate the thickness of the intestinal mucus layer, the production of goblet cells, and the expression of both mucins. Further, the expression of one of the major mucins secreted in the lumen of the colon by the goblet cells, Mucin-2, using immunohistochemistry (IHC) (Figures 5B, D). The examination of both staining PAS and AB revealed severe damage of the gut barrier with the thinness of the mucus layer as a result of the loss and the reduction of the formation of goblet cells which directly impact mucus in the model group compared to the normal control group, there was a decrease in mucus production. Conversely, CCP significantly increased goblet cell formation and mucin expression, leading to improved mucus layer thickness in CCPL and CCPH. The results of the polysaccharide treatment groups showed a close similarity with the normal control group. Thus, it suggests that CCP could improve the regeneration of the mucus layer in the colon of mice treated only with DSS. Moreover, as shown in Figure 5B, these first results were confirmed with immunohistochemistry (IHC) staining; in the model group, the DSS treatment markedly reduced the expression of Mucin-2 compared to the normal control tissue, while the expression of Mucin-2 in CCPH and CCPL was higher in a dose-dependent manner compared to DSS group. Mucin-2 expression was close in both groups, CCPH and NC. The conservation of the mucus layer was consistent with the observed results in CCP treatment. As evidenced by these findings, CCP may exert a protective effect in DSS-induced colitis in mouse models by enhancing mucus thickness through increased goblet cell numbers and mucin production, particularly Mucin-2.

### 3.6 CCP enhanced barrier function and tight junction protein expression

**FIGURE 6:**
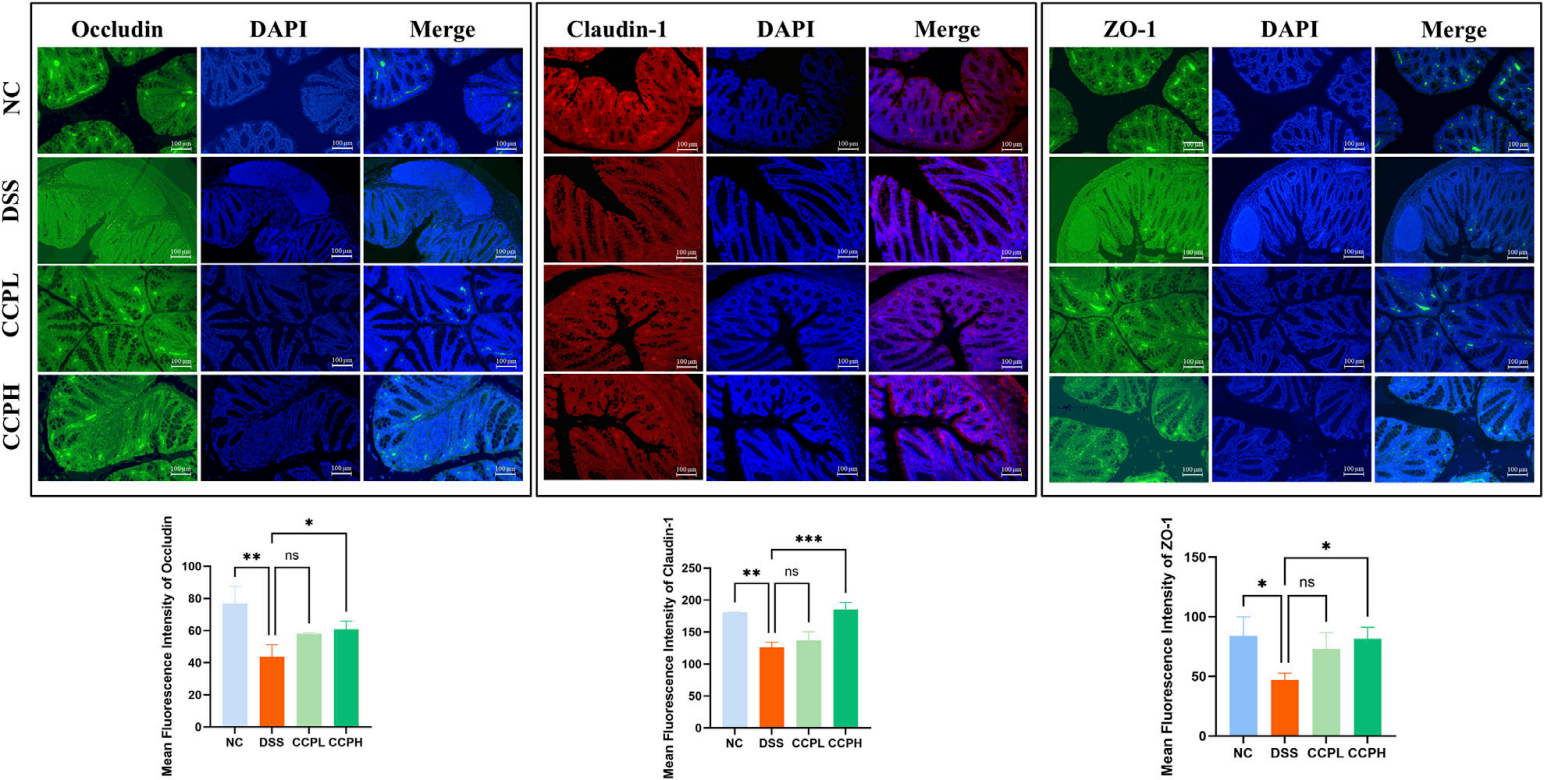
CCP enhances the expression level of tight junction proteins in colon tissue, as observed through Immunofluorescent (IF) analysis for Occludin, Claudin-1, and ZO-1 proteins at a magnification of ×20. And The Quantification Graph of each Immunofluorescent staining. Results: ns (not significant), *p < 0.05, **p < 0.01, ***p < 0.001.

To check the therapeutic impact of CCP on tight junction protein expression and intestinal integrity, immunofluorescent staining was performed. Colitis compromises the colon barrier and increases intestinal permeability. As depicted in Figure 6, DSS causes significantly decreased levels of ZO-1, Claudin-1, and Occludin in the colon tissue of the DSS group compared to the NC group, indicating compromised intestinal barrier function. In contrast, treatment with CCP at both low and high doses notably reversed this reduction in a dose-dependent manner. These results demonstrate that CCP supplementation helps maintain intestinal epithelial barrier integrity by supporting the expression of tight junction proteins.

### 3.7 CCP modulates cytokines mRNA expression in colon tissue

**FIGURE 7:**
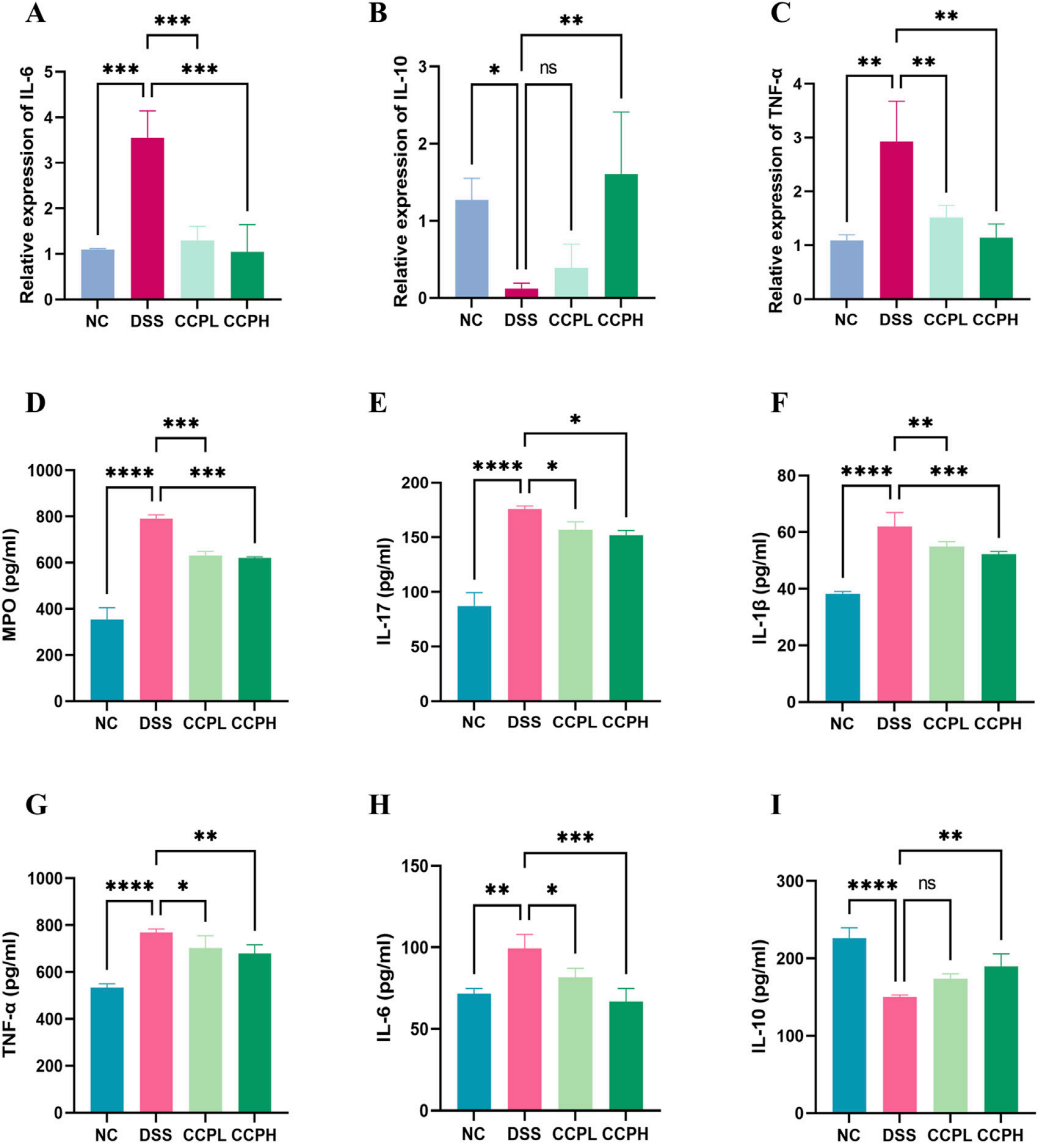
CCP Regulates the Balance of Pro-inflammatory and Anti-inflammatory Cytokine’s Expression and Secretion in Colon Tissue. Panels **(A–C)** show the mRNA expression levels of IL-6, IL-10, and TNF-α. Panels **(D–I)** display secretion levels (ELISA) of MPO, IL-17, IL-1β, TNF-α, IL-6, and IL-10. Results are presented as ns (not significant), * (*p* < 0.05), ** (*p* < 0.01), *** (*p* < 0.001), **** (*p* < 0.0001).

In this study, the impact of DSS treatment on cytokine expression was evident, with significant increases in pro-inflammatory cytokines IL-6 and TNF-α mRNA levels (*p* < 0.001 and *p* < 0.01, respectively), along with a notable decrease in anti-inflammatory cytokine IL-10 mRNA expression (*p* < 0.05) in the model group compared to the normal control group. Interestingly, administration of CCP led to a dose-dependent effect on cytokine expression in mice, resulting in a significant upregulation of IL-10 mRNA levels (*p* < 0.001 for CCPH group) and downregulation of IL-6 (*p* < 0.001 for both CCPL and CCPH groups) and TNF-α (*p* < 0.01 for both CCPL and CCPH groups) mRNA levels compared to the DSS group. These findings underscore CCP’s potential to modulate cytokine expression, enhancing anti-inflammatory responses while inhibiting pro-inflammatory responses, thus suggesting its efficacy in regulating and alleviating the inflammatory response (Figures 7A–C).

### 3.8 Effect of CCP on the secretion of inflammation-related cytokines in colon tissue

Furthermore, the immunomodulatory impact of *C. cibarius* Fr.-derived polysaccharide was evaluated by measuring the expression levels of both pro-inflammatory and anti-inflammatory cytokines using ELISA. In colon tissue, the DSS group exhibited a significant increase in pro-inflammatory cytokines IL-17 (*p* < 0.0001), IL-1β (*p* < 0.0001), TNF-α (*p* < 0.0001), and IL-6 (*p* < 0.01), compared to the NC group (Figures 7D–I). In contrast, both doses of CCP notably reduced the levels of these pro-inflammatory markers in a significant manner in the treatment groups CCPL and CCPH: IL-17 (*p* < 0.05 for both treatment groups), IL-1β (*p* < 0.01 and *p* < 0.001, respectively), TNF-α (*p* < 0.05 and *p* < 0.01, respectively), and IL-6 (*p* < 0.05 and *p* < 0.001, respectively) compared to the DSS group. Additionally, in the model group, DSS treatment significantly decreased the levels of the anti-inflammatory cytokine IL-10 (*p* < 0.0001) compared to the normal control group, while CCP significantly enhanced the expression of these anti-inflammatory cytokines in colon tissue, particularly in the high-dose group compared to the model group (*p* < 0.01) (Figures 7D–I). These findings indicate that CCP may exert a protective effect by attenuating the inflammatory response in the colon.

### 3.9 Effect of CCP on neutrophil infiltration

Myeloperoxidase (MPO) serves as a marker for neutrophil infiltration in colon tissue following colitis induction. As depicted in Figure 7D, colon tissue from the DSS group exhibited a significant increase in MPO activity compared to the NC group (*p* < 0.0001). In contrast, administration of CCP significantly reduced MPO secretion in the colon tissue of both CCP-treated groups CCPH and CCPL (*p* < 0.001 for both treatment groups), approaching levels observed in the NC group. These findings suggest a potential suppressive effect of CCP on neutrophil infiltration, indicated by the decreased MPO activity.

### 3.10 CCP regulates the macroecology of gut microbiota

**FIGURE 8:**
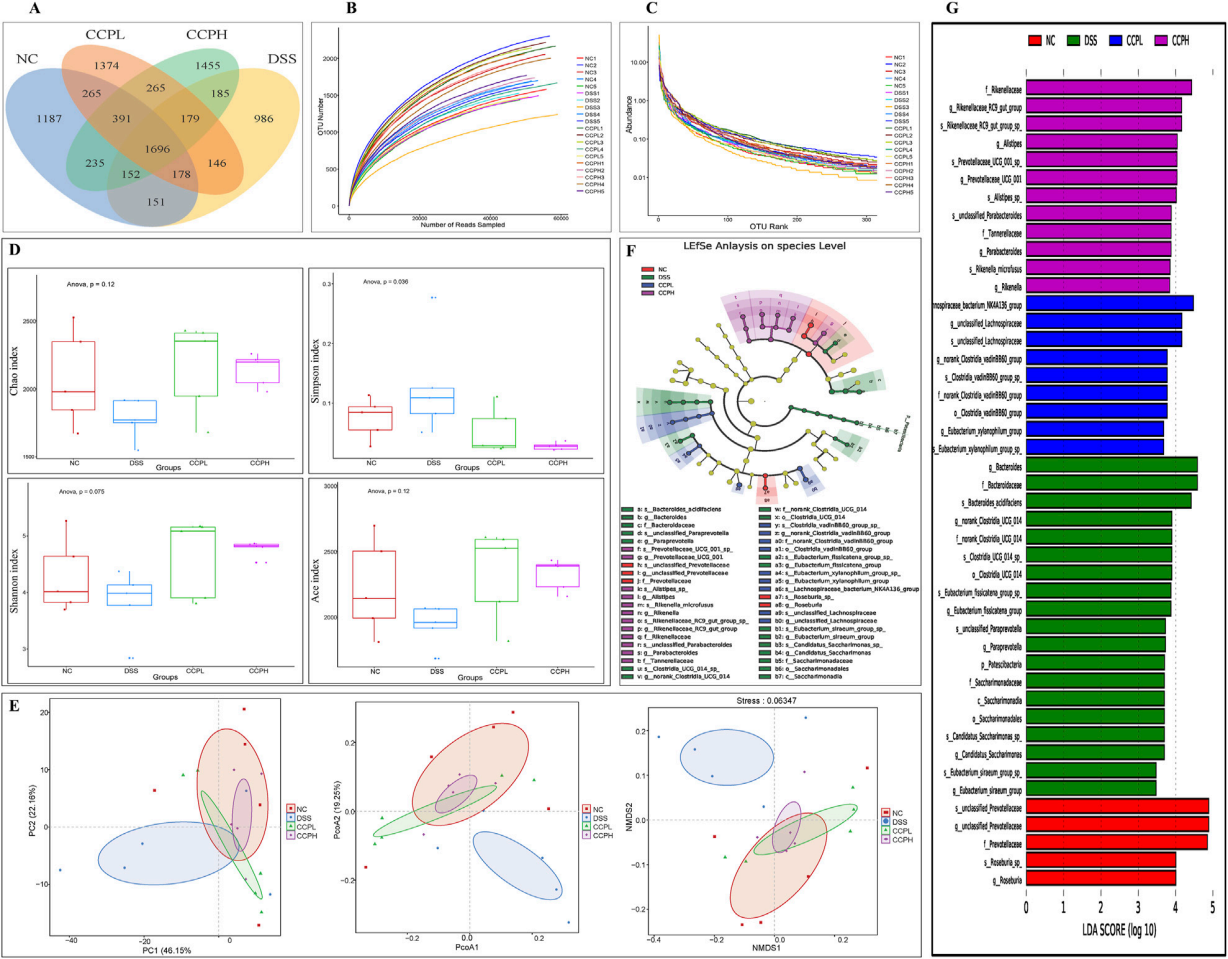
CCP alleviates the DSS-induced dysbiosis. **(A)** The Operational Taxonomic Units Venn diagram illustrating the shared OTUs across the different experimental; **(B)** Rarefaction curve Rank: gives an insight into the diversity within the samples; **(C)** Rank-abundance curve illustrating the relative abundance of microbial taxa across the different experimental groups; **(D)** Alpha diversity indices to assess richness, abundance, and diversity: Chao1, Simpson, Shannon and ACE indices; **(E)** Beta diversity evaluation to assess the microbial community structure across the groups. through Principal Coordinates Analysis (PCoA), Principal Component Analysis (PCA), and Non-metric Multidimensional Scaling (NMDS), **(F)** The cladogram of Linear Discriminant Analysis Effect Size (LEfSe) analysis; **(G)** Linear Discriminant Analysis (LDA) scores in differentially abundant taxa (LDA >4.0).

To explore the prebiotic effect of CCP on the composition and diversity of intestinal microbiota in a DSS-induced colitis mouse model. 16s rRNA sequencing was employed to analyze the V3-V4 region from the fecal samples. Venn diagram (Figure 8A) provides insight into the impact of disease and subsequent treatment CCP on the gut microbiome composition by analyzing the number of Operational Taxonomic Units (OTUs) unique to and shared between the study groups. The analysis revealed distinct patterns in group samples, the DSS group had the lowest number of unique OTUs (986) compared to NC (1,187) and the two treatments groups, CCPL and CCPH, suggesting a loss of microbial diversity in the model group. Furthermore, the analysis indicates that the CCPH group has a greater number of OTUs, 1,455, than the CCPL group, with 1,374 OTUs. Interestingly, the two treatment groups, CCPL and CCPH, displayed a greater overlap with the NC group compared to the overlap of the DSS and NC groups; this suggests that the microbial community of the treatment groups CCPL and CCPH is more similar to the normal control group. This may indicate an effective restoration of the gut microbiome towards a healthier state, potentially resembling the NC group. The OTU rarefaction (Figure 8B) and the Abundance rank curves (Figure 8C) provide insights into the alpha diversity and relative richness of the microbial communities across the groups. The rarefaction curves revealed that the NC group exhibited the highest number of observed OTUs and the greatest diversity among the three groups. In contrast, the DSS group displayed a distinct pattern with curves leveling off at a comparatively lower number of observed shared OTUs, indicating the lowest overall microbial diversity compared to the NC and CCP treated groups. However, both CCPH and CCPL samples showed higher rarefaction curves and greater OTU richness than the DSS group, indicating increased microbial diversity. Figure 8D results revealed that the DSS-treated group exhibited reduced species richness (Ace and Chao1 indices) and diversity (Shannon and Simpson indices) compared to the normal group, indicative of gut microbiome dysbiosis with decreased bacterial abundance and diversity in the model group. However, the supplementation of CCP showed significant changes in the microbiome composition, CCPL group’s noticeable trend towards increased Simpson, Shannon, Chao1, and Ace indices was observed. Also, the CCPH group showed a significant increase in Chao 1 and Ace indices compared to the DSS group, these results indicate that CCP supplementation reversed the DSS-induced dysbiosis by restoring the Alpha diversity indices (Figure 8D). A significant increase is observed in the Shannon Index, demonstrating a significant restoration of diversity in CCPH. In summary, these findings demonstrate that the DSS-induced colitis model significantly disrupts gut microbiome diversity and richness. Nevertheless, treatment with CCP appears to restore these alpha diversity parameters in a dose-dependent manner. Beta diversity analysis was employed to characterize the gut microbiome structure and to reveal compositional similarities or dissimilarities between the experiment groups. To further evaluate the prebiotic potential effect of CCP on DSS-induced dysbiosis, the Beta diversity parameters including Principal Coordinates Analysis (PCoA) with weighted UniFrac distance, Principal Component Analysis (PCA), and Non-Metric Multidimensional Scaling (NMDS) were analyzed (Figure 8E). The results demonstrate that the four different groups (NC, DSS, CCPL, and CCPH) are visibly separated from each other. The PCoA plot analysis shows the DSS group positioned separately from the NC group; this latter is also positioned separately from the other three groups, suggesting that the microbial profile of the normal control is distinct from the colitis model and the two treatment groups (CCPL and CCPH), it also implies that the microbial community composition is most altered in the disease model compared to the normal control indicating a significant impact of DSS-induced colitis on the gut microbiota. However, the profiles of the treated groups, CCPL and CCPH, are positioned closer to each other than to the DSS group, indicating that the microbial profiles of these two groups are more similar to each other than to the disease model and show a partial shift towards the control group, suggesting the ability of CCP to restore a healthier gut microbiota. Importantly, the PCA plot and NMDS reinforce the observations from PCoA, depicting distinct clusters for each group.

Linear Discriminant Analysis Effect Size (LEfSe) and Linear Discriminant Analysis were performed to investigate and identify the impact of colitis and the different treatments on the potential biomarkers that contribute to differences in the microbiome composition of the four groups (Figures 8F, G). The key markers in the Normal control were *unclassified Prevotellaceae* (Family: *Prevotellaceae*, Genus: *unclassified Prevotellaceae* and species: *unclassified Prevotellaceae*) followed by *Roseburia* (Genus: *Roseburia* and species: *Roseburia sp*). Nonetheless, the model group is characterized by an expansion of *Bacteroides* (family: *Bacteroidaceae*, genus: *Bacteroides* and species: *Bacteroides acidifaciens*); this expansion is a hallmark of the dysbiosis observed in the DSS group. However, the CCP treatment appears to have an impact on modulating the gut microbiome; CCPL is highly enriched with *Lachnospiraceae bacterium NK4A136 group* (genus: *unclassified Lachnospiraceae* and species: *unclassified Lachnospiraceae*) and *norank Clostridia vadinBB60 group* (family: *norank Clostridia* *vadinBB60 group*, genus: *norank Clostridia vadinBB60 group* and species: *Clostridia vadinBB60 group sp*). While the key markers in CCPH were: *Rikenellaceae* (family: *Rikenellaceae*, genus: *Rikenellaceae* *RC9 gut group* and species: *Rikenellaceae RC9 gut group sp*) and *Alistipes* (genus: *Alistipes* and species: *Alistipes sp*).

### 3.11 CCP remolds the microecology of the gut microbiota and CCP effects on different levels of microbial abundance

**FIGURE 9:**
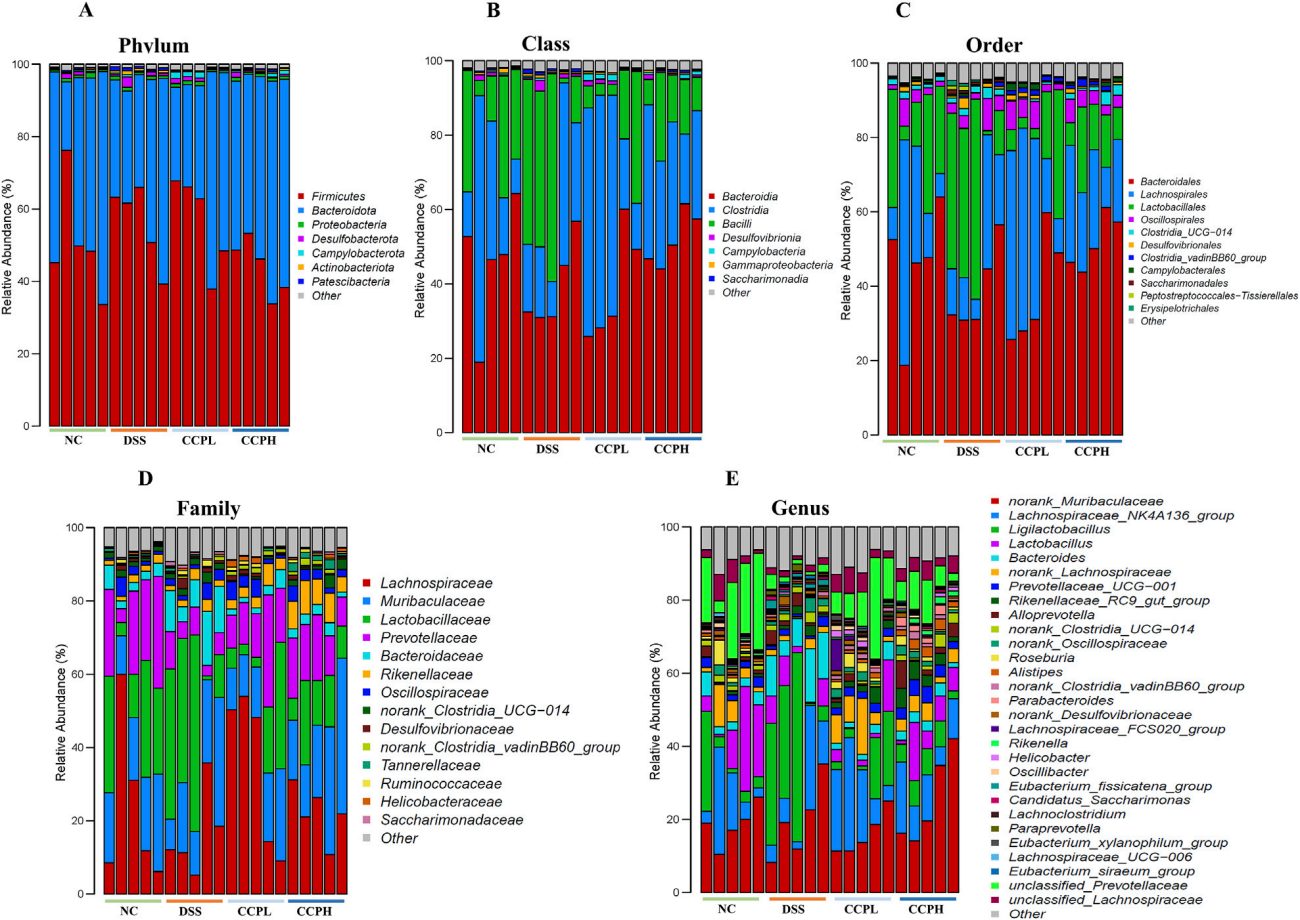
Modulation of gut microbiota ecology by CCP. **(A)** display microbial abundance at the phylum level, **(B)** shows microbial abundance at the class level, while **(C–E)** detail abundance at the order, family, and genus levels, respectively.

Bacterial diversity was further examined at different taxa levels. Figures 9A–E, present the relative abundances of different bacterial taxa from the phylum to the genus taxonomic levels to visualize the distribution of the intestinal microbiota in the experimental groups; it showed a consistent pattern of microbiome changes across different taxonomic levels. The distribution at the phylum level in the healthy group NC, the model one DSS, and both treatment groups with Low and High doses of CCP are depicted in Figure 9A; Supplementary Table S3. The most observed phyla in the four groups were *Firmicutes*, *Bacteroidota*, *Proteobacteria*, and *Desulfobacterota*, but their relative abundances differed. Consistent with previous findings, *Firmicutes* and *Bacteroidota* were the dominant phyla identified in all groups. As Figure 9A; Supplementary Table S3 show, the predominant phyla in the normal control group were *Firmicutes* (51.16%), *Bacteroidota* (45.45%), and *Desulfobacterota* (0.62%), with a higher proportion of *Firmicutes* than *Bacteroidota*, exhibiting the closest balance between these two dominant phyla. Compared with the NC group, the proportions shifted with a slight increase in *Firmicutes* (56.38%), *Desulfobacterota* (1.05%), and a decrease in *Bacteroidota* (39.14%) for the DSS group, interestingly this group exhibited a higher relative abundance of *Firmicutes* among all the groups. However, in the experimental CCP treatment groups, CCPL showed percentages of *Firmicutes* (56.54%), *Bacteroidota* (38.99%), and *Desulfobacterota* (0.84%). In CCPH, there was a noticeable decrease in *Firmicutes* (43.77%) and *Desulfobacterota* (0.62%), along with an increase in *Bacteroidota* (52.25%) compared to the DSS group. At the class level (Figure 9B; Supplementary Table S4), variations in the relative abundance of bacterial classes were observed among the groups. The bar plot illustrates the seven most abundant microbial classes, dominated by *Bacteroidia*, *Clostridia*, and *Bacilli*, with other classes such as *Desulfovibrionia* and *Campylobacteria* present in lower abundances. The normal control exhibited a balanced microbiome composition, with *Bacteroidia* being the most abundant class (45.4%), followed by *Clostridia* (29.9%) and *Bacilli* (20.9%). In contrast, the disease model showed a shift in microbial profile, with increased *Bacilli* (31.8%) and decreased *Clostridia* (24.0%) and *Bacteroidia* (39.1%), alongside slight increases in *Desulfovibrionia* compared to the NC group, indicating dysbiosis. The low-dose treatment group displayed a different microbial profile with increased *Clostridia* (43.2%), decreased *Bacilli* (13.0%) and *Desulfovibrionia* (0.83%), and a similar abundance of *Bacteroidia* (39.0%) compared to the DSS group. Remarkably, the high-dose treatment group (CCPH) showed significant restoration of gut microbiome composition, with increased *Bacteroidia* (52.2%) and *Clostridia* (29.9%), and decreased *Bacilli* (13.5%) and *Desulfovibrionia* (0.62%), resembling the balanced profile of the normal control group. Analysis at the order and family taxonomic levels (Figures 9C, D), confirmed the previously observed variations in the relative abundance of bacterial taxa. Analysis at the genus level (Figure 9E) revealed notable differences in gut microbiome composition between the healthy control (NC) and DSS-treated groups. Compared to the NC group, the DSS group displayed a dysbiotic profile, including a marked increase in the relative abundance of *Bacteroides* and *Ligilactobacillus* and a decrease in the relative abundance of beneficial taxa such as *Lachnospiraceae NK4A136 group*, *norank* *Lachnospiraceae*, *Lactobacillus*, *Prevotellaceae UCG-001*, and a great decrease in the relative abundance of *Roseburia* and *Alistipes*. Interestingly, CCP reversed some of the DSS-induced changes. Compared to the DSS group, the CCPL group revealed a lower relative abundance of the most abundant genera of the model group with an increase in *Lachnospiraceae NK4A136 group* and *unclassified Prevotellaceae*. Interestingly, CCPH demonstrated a shift from the DSS-treated group, resembling the balanced profile observed in the normal control group. The abundance of *norank Muribaculaceae*, *Lachnospiraceae NK4A136 group*, *Lactobacillus* and *unclassified Prevotellaceae* were enriched, while the levels of *Ligilactobacillus* and *Bacteroides* decreased. The analysis of the composition of the gut microbiome at different taxonomic levels reveals the potential effects of the disease on the gut microbiome, the DSS group likely represents a dysbiotic state compared with the NC, and both treatment groups, CCPL and CCPH, exhibited a trend towards a reversal of this shift, especially the high dose one, this suggests that the high-dose treatment (CCPH) had a more pronounced effect on the gut microbiota compared to the low-dose treatment (CCPL), shifting the balance between the dominant phyla *Firmicutes* and *Bacteroidota* closer to the normal control levels, mitigating the dysbiosis.

## 4 Discussion

DSS is a widely utilized agent for inducing a colitis model, exhibiting morphological and symptomatic similarities to ulcerative colitis (UC) in humans to study the pathways and the potential of different treatments in managing this illness (Randhawa et al., 2014). The mice were given drinking water containing 3% DSS for 7 days to induce the UC model. The presence of symptoms like diarrhea, significant body weight reduction, and rectal bleeding in mice treated with DSS indicate the successful induction of a colitis model. Drug research is turning to natural products, extracts (Guo et al., 2017; Liu et al., 2022), especially prebiotics such as edible mushrooms, plants, and seaweed polysaccharides (Li et al., 2021), due to their diverse range of bioactivities and ability to treat IBD through regulation of the immune response and gut microbiota (Zhou et al., 2023).

The present study focuses on crude polysaccharides extracted from *C. cibarius* Fr., a wild mushroom with consistently documented antioxidant, antimicrobial, and anti-inflammatory properties (Sevindik, 2019; Kolundžić et al., 2017; Nasiry et al., 2017), and investigates its therapeutic effect for the first time on the different inflammatory symptoms of ulcerative colitis, colon injury, immune disorder, and gut microbiota dysbiosis. The *C. cibarius* Fr. polysaccharides (CCP) were extracted using hot water, and the monosaccharide composition of CCP was analyzed through HPLC. As previously reported, Glucose, Galactose, Mannose, Ribose, and Glucuronic acid were the main components (Zhou et al., 2023).

Following the development of DSS-induced colitis and the introduction of CCP as a potential treatment, the clinical symptoms of UC were significantly alleviated, as described by the Disease Activity Index, taking into consideration body weight loss, rectal bleeding, and changes in stool consistency, compared to the model group, these preliminary findings are in agreement with a previous study that reported the ability of *Smilax china* L. polysaccharide in alleviating clinical symptoms of colitis (Li et al., 2023). Additionally, higher dosage of CCP was more effective in alleviating clinical symptoms.

The intestinal tract is a complex and dynamic ecosystem in which the host and the microbiome engage in a delicate dialogue to maintain homeostasis. The host’s defense pathways, comprising innate and adaptive immune systems play a pivotal role in recognizing and responding to potential threats, such as pathogenic microbes. Dysregulation of this balance can promote the development of inflammatory bowel diseases (Friedrich et al., 2019). The thymus is a key lymphoid organ (Luo et al., 2021) with spleen, are the main immune organs (Yu and Zheng, 2019), and have a crucial role in immune cells’ development, maturation, and regulation. Their size is linked with the inflammatory condition. For instance, thymus atrophy and spleen enlargement are commonly observed in inflammatory disorders, reflecting the dysregulation of the immune response. As reported by previous research, DSS treatment significantly decreased the thymus index and increased the spleen index (Meng et al., 2022) and caused a severe shrinkage of the colon and significantly reduced colon and small intestine indices compared to the normal control group (Ullah et al., 2023), however, CCP-treated groups (CCPH and CCPL) showed increases in colon length, small intestine, colon, and thymus indices, while the spleen index was reduced. These results indicate CCP’s anti-inflammatory and immune-modulatory potential, with a more pronounced ameliorative effect observed for the higher CCP dose.

Additionally, the intestinal barrier is a complex defense and multi-layered system within the gastrointestinal tract that includes a mechanical intestinal barrier, immune barrier, and microbial barrier (Ji et al., 2024). The mechanical intestinal barrier, a crucial physical and functional barrier, is composed of mucus layers and intestinal epithelial cells, tightly connected by tight junctions’ protein, creating a selective barrier that regulates the passage of nutrients, water, and electrolytes while restricting the entry of potentially harmful substances like bacteria and toxins. The mucus layer which is the most outer layer and is mainly composed of water (>98%) and mucin-2 produced by goblet cells, is essential for maintaining and protecting the underlying intestinal epithelial cells. (Stolfi et al., 2022; Di Tommaso et al., 2021). Recent studies found a noticeable link between impaired intestinal barrier functions and the onset of colitis. This is marked by leaky gut symptoms like increased cell infiltration in colonic mucosa and a decline in the number of goblet cells (Yu et al., 2024; Dorofeyev et al., 2013). Consuming DSS leads to colonic tissue damage, disrupting the mucosal structure, resulting in leaky gut, decreased intestinal integrity, and loss of goblet cells (Xiang et al., 2021; Chen et al., 2020; Chami et al., 2018). Our study exhibited similar results, showing the DSS effect on the colon leads to damage, disruption of mucosal structure, decrease in the expression of tight junction proteins, and also goblet cell depletion. Nevertheless, CCP supplementation demonstrated a dose-dependent potential therapeutic effect, significantly ameliorating all these pathological manifestations and restored the normal and healthy colonic architecture in both treatment groups. These findings are following previously published work (Liu et al., 2015), that investigated the therapeutic effect of different compounds on DSS-induced colitis.

Tight junction proteins are vital components of the cell adhesion complexes that distinguish between apical and basolateral membrane domains. These are responsible for maintaining the cell polarity and regulating the selective diffusion of the molecules across membranes (Shin et al., 2006). These proteins are also valuable predictive markers for assessing the severity of colitis. DSS is known to damage the tight junction proteins. However, supplementation with CCP has been shown to reverse these detrimental effects. These results are consistent with a previous study (Xu et al., 2023), where *Scorias spongiosa* polysaccharide restored the damage of tight junction proteins induced by DSS. Taken together, these findings provide evidence that CCP supplements, particularly at higher dose, effectively counteracted the pathological changes induced by DSS and restored the colonic barrier.

Recent advancements in genetics and immunological research found that cytokines are considered to be strongly linked to the etiology and progression of Crohn’s disease and colitis. Previous studies demonstrated the role of cytokines in controlling intestinal inflammation and clinical manifestations of IBD. Pro-inflammatory cytokines such as IL-17, IL-1β, IL-6, and TNF-α have been identified as the key driver in colitis-associated inflammation (Xiang et al., 2021). On the other hand IL-10 acts as an anti-inflammatory cytokine that plays a crucial role in regulating immune response by decreasing the expression of inflammation and restoration of intestinal homeostasis through mucus metabolism (Di Tommaso et al., 2021). In this work, we determined the expression level pattern of the pro-inflammatory and anti-inflammatory markers in the context of DSS-induced colitis. Our findings exhibit a notable imbalance in cytokines in the model group. However, CCP supplementation resulted in a dose-dependent reduction in the expression of pro-inflammatory cytokines and increase in anti-inflammatory cytokines, suggesting its immunomodulatory effect in restoring intestinal immune balance.

The intestinal microflora, referred to as the “forgotten inner organ” performs a multitude of critical structure, defensive, and metabolic roles that are important for the host’s health. Gut microbiota helps in digesting complex and indigestible substances like polysaccharides to get essential nutrients. Additionally, the gut microbiome produces vital vitamins like K and B which help against infections (Clarke et al., 2014; Schirmer et al., 2019; Alsholi et al., 2023). Imbalance and disruption of the gut microbiota have been associated with the onset of different diseases such as IBD, obesity, diabetes, and social isolation or anxiety (Ritchie et al., 2017).

Previously published work showed that diversity and richness within disease groups indicated an imbalance, with significant decline and dysbiosis in the gut microbiota (Khan et al., 2019). An increase of pro-inflammatory microbial species compared to anti-inflammatory ones is a common indicator of low microbial diversity in the gut microbiome and this imbalance leads to the production of inflammatory mediators like lipopolysaccharides and other molecules derived from microbes, which can disrupt the intestinal epithelial and also mucosal barrier dysfunction (Ramirez et al., 2020). Previous work showed that the predominant phyla present in the gut are *Bacteroidetes*, *Proteobacteria*, *Firmicutes*, and *Actinobacteria* (Eckburg et al., 2005). Conferring to this, the results demonstrate that *Bacteroidetes* and *Firmicutes* are the predominant phyla.

The current work explores the prebiotic effect of CCP in the context of DSS-induced dysbiosis. Our results showed that the DSS group exhibited a significant decline in the abundance and richness of bacterial diversity. The model group formed distinct clusters and separated from the normal control group. Conversely, the CCP treatment reversed this dysbiosis particularly at the higher dose by increasing the richness and was found more similar to the normal control showing a promising prebiotic potential effect. These results are supported by previously published work (Kanwal et al., 2020) which investigates the treatment effect of *Dictyophora indusiata* polysaccharide on DSS-induced colitis. *Prevotellaceae* and *Roseburia*, known as tract residents, were predominant in the normal control group; *Roseburia* plays a vital role in digestion, immune function, and maintaining a healthy gut barrier. However, colitis is characterized by several key disruptions according to a previous study: the DSS group is characterized by a great reduction in *Roseburia* (Nie et al., 2021) and an increase in the opportunist pathogens and mucin degrader *Bacteroides, B. acidifaciens* (Chang et al., 2021; Pan et al., 2022; Thipart et al., 2023). Nevertheless, CCP supplementation contributes to a healthier gut environment. It exerts a twofold effect on the gut microbiota: increasing its diversity and modulating its composition. In the low-dose treatment, the *Lachnospiraceae bacterium NK4A136* group was enriched; this bacterium is known as butyrate-producing bacteria (Thipart et al., 2023), while the high-dose treatment was enriched with *Rikenellaceae RC9 gut group sp* and *Alistipes* sp. This study, (Haiou et al., 2024), correlate the increase of the expression of MUC-2 and ZO-1 and occludin in colon tissue with the increase of the following bacteria *Alistipes,* *Lachnospiraceae NK4A136 group*, and *Lachnospiraceae UCG006*, and the decrease in *Bacteroides*. Additionally, these results align with previous research, where *Chrysanthemum morifolium* polysaccharide ameliorates colitis by the expansion of beneficial bacteria such as *Lachnospiraceae*, *Rikenellaceae*, and *Lactobacillus* by increasing the release of several anti-inflammatory factors such as IL-4 and IL-10 and reducing the release of pro-inflammatory cytokines such as IL-23, TNF-α, IL-1β and IL-6, from the harmful bacteria such as *Bacteroides* (Tao et al., 2017). Moreover, *Alistipes* is known to reduce intestinal inflammation and promote intestinal maturation, consequently improving the intestinal mucosa (Xiang et al., 2021).

In summary, this study suggests that treatment with *C. cibarius Fr.* Polysaccharide as a crude extract may be effective in alleviating the symptoms of DSS-induced colitis. Specifically, it appears to contribute to the restoration of intestinal barrier integrity, modulation of the gut microbiota microecology, regulation of immune responses, and reduction of inflammation.

## 5 Conclusion

The study examined the effect of the crude extract of polysaccharide from the mushroom *C. cibarius* Fr., (CCP) on a model of inflammatory bowel disease, ulcerative colitis, using DSS. The different results suggest that the crude polysaccharide extract was able to significantly alleviate the symptoms of colitis in the mice. CCP possesses multifaceted effects on the intestinal barrier integrity, the inflammatory cytokine profiles, and the gut microbiome composition. Specifically, these findings indicate that the crude polysaccharide extract from the golden chanterelle mushroom, *C. cibarius* Fr., helped to restore the integrity of the intestinal barrier, which is often compromised in inflammatory bowel conditions, ameliorate the inflammatory conditions in the colon, and reverse the dysbiosis occurring in the gut microbiome by reshaping its composition.

## Data Availability

The raw data supporting the conclusions of this article will be made available by the authors, without undue reservation.
