# Supplementary material for: Polysaccharides derived from golden mushroom (Cantharellus cibarius Fr.) modulate gut microbiota and enhance intestinal barrier function to ameliorate dextran sulfate sodium-induced colitis in mice
Source: Front Pharmacol. 2024 Dec 18;15:1498625. doi: 10.3389/fphar.2024.1498625 (PMC11688367; doi:10.3389/fphar.2024.1498625)
Supplement: Supplementary file 1 [file Table1.pdf]

Polysaccharides Derived from Golden Mushroom (*Cantharellus cibarius* Fr.) Modulate Gut Microbiota and Enhance Intestinal Barrier Function to Ameliorate Dextran Sulfate Sodium-Induced Colitis in Mice

Yamina Alioui[1], Hidayat Ullah[1], Sharafat Ali[2], Mujeeb Ur Rahman[1], Maroua Elkharti[2], Nabeel Ahmed Farooqui[1], Ata Ur Rehman[3], Muhammad Ilyas[1], Duaa M. Alsholi[4], Nimra Zafar Siddiqi[1], Muhsin Ali[1], Liang Wang[5]*, and Yi Xin[1]*,

[1]Department of Biotechnology, College of Basic Medical Science, Dalian Medical University, Dalian 116044, China.

[2]Department of Biochemistry and Molecular Biology, College of Basic Medical Science, Dalian Medical University, Dalian 116044, China.

[3]Multidisciplinary Neuroprotection Laboratories, Duke University School of Medicine, Durham, NC 27708, USA.

[4]Department of Medical Laboratories Sciences, Faculty of Allied Medical Sciences, Zarqa University, Zarqa, Jordan.

[5]Stem Cell Clinical Research Center, National Joint Engineering Laboratory, Regenerative Medicine Center, The First Affiliated Hospital of Dalian Medical University, Dalian 116011, China.

***Correspondence authors**: Liang Wang; E-mails address: wangliang@dmu.edu.cn, Yi Xin; E-mails address: xinyi412@DMU.edu.cn

Supplementary Tables:

Table S1. Antibodies used in IHC and IF.

Antibody target	Antibody type	Antibody dilution	Catalog Number	Company
Mucin-2	Polyclonal	1:1000	27675-1-AP	Proteintech
ZO-1	Polyclonal	1:1000	21773-1-AP	Proteintech
Claudin-1	Polyclonal	1:1000	13050-1-AP	Proteintech
Occludin	Polyclonal	1:400	27260-1-AP	Proteintech

Table S2. List of primer sequences (5' to 3’) employed for assessing mRNA expression level.

Gene	Forward primer	Reverse primer	Concentration	Supplier
IL-6	TACCACTTCACAAGTCGGAGGC	CTGCAAGTGCATCATCGTTGTTC	10uM	Thermo Fisher Scientific
IL-10	CGGGAAGACAATAACTGCACCC	CGGTTAGCAGTATGTTGTCCAGC	10uM	
TNF-α	GGTGCCTATGTCTCAGCCTCTT	GCCATAGAACTGATGAGAGGGAG	10uM	
β-Actin	ATCGCTGCGCTGGTCG	GTCCTTCTGACCCATTCCCA	10uM	

Table S3. Percentage Composition of Bacterial Phylum Across the Different Experimental Groups.

**Phylum**	**NC (%)**	**DSS (%)**	**CCPL (%)**	**CCPH (%)**
Firmicutes	51.16498	56.38444	56.53569	43.76898
Bacteroidota	45.45044	39.14335	38.98835	52.25313
Desulfobacterota	0.616557	1.047194	0.840376	0.621337
Campylobacterota	0.253288	0.226369	1.028054	0.561322
Other	2.514738	3.198646	2.607525	2.795233

Table S4. Relative Abundance of Bacterial Class Across the Different Treatment Groups.

Class	NC (%)	DSS (%)	CCPL (%)	CCPH (%)
Bacteroidia	45.42748	39.13088	38.96476	52.23077
Clostridia	29.91209	24.04955	43.16954	29.91009
Bacilli	20.89925	31.79561	12.91492	13.52232
Desulfovibrionia	0.610632	1.040769	0.833116	0.617807
Campylobacteria	0.253288	0.226369	1.028054	0.561322
Other	2.897263	3.756821	3.089606	3.15768
